# A qualitative study on the needs of cancer caregivers in Vietnam

**DOI:** 10.1080/16549716.2021.1961403

**Published:** 2021-08-26

**Authors:** Chris Jenkins, Hien Thi Ho, Hoa Phuong Le Nghiem, Gillian Prue, Lynne Lohfeld, Michael Donnelly, Minh Van Hoang, Olinda Santin

**Affiliations:** a Centre for Public Health, Queen’s University Belfast, Belfast, UK; b Faculty of Medicine, Hanoi University of Public Health, Hanoi, Vietnam; c Vietnam Cancer Hospital, Hanoi, Vietnam; d School of Nursing and Midwifery, Queen’s University Belfast, UK

**Keywords:** Cancer, carergivers, Global Health, Vietnam, qualitative

## Abstract

**Background:**

Vietnam has experienced a rapid increase in cancer incidence with many cancers (70%) being diagnosed at a late stage. The majority of physical and psychosocial care is provided by caregivers with minimal professional input. Due to limited resources in hospitals and social and cultural norms regarding caregiving in Vietnam, caregivers provide a range of supportive functions for family members diagnosed with cancer.

**Objectives:**

This study sought to provide empirical evidence on the self-identified unmet needs of caregivers of inpatients in national oncology hospitals in Vietnam.

**Methods:**

Focus groups and in-depth interviews were conducted with caregivers (n = 20) and health care providers (n = 22) in national oncology hospitals in Hanoi and Ho Chi Minh City. Data was collaboratively analysed using thematic analysis. Findings were validated through key stakeholder group discussions with both caregivers and healthcare providers across multiple regions in Vietnam.

**Results:**

Analysis demonstrated that the burden of informal care is high with many caregivers managing patient’s severe and complex health needs with minimal support. Caregivers highlighted four main areas of critical need: (i) challenges in providing long term care, particularly in hospital and in-patient settings, such as accessing comfortable facilities, accommodation and finance; (ii) information needs about cancer, treatment, and nutrition; (iii) support for the emotional impact of cancer; and (iv) training about how to provide care to their family members during treatment and recovery phases.

**Conclusions:**

Caregivers provide invaluable support in supporting people with a cancer diagnosis, particularly given wider systemic challenges in delivering cancer services in Vietnam. Increasing visibility and formal support is likely to have both a positive impact upon the health and wellbeing of caregivers, as well as for cancer patients under their care. Given its absence, it is critical that comprehensive psychosocial care is developed for caregivers in Vietnam.

## Background

There are an estimated 165,000 new cancer diagnoses and 115,000 cancer-related deaths in Vietnam per year [1]. The most common cancers associated with mortality across both sexes are liver (23.48%), lung (19.14%), stomach (13.92%), and breast (5.64%). Research on cancer service delivery in Vietnam is limited, but indicates that there are significant challenges across the health system in providing quality cancer services [2–6]. Oncology hospitals and healthcare workers are often under resourced and significantly over stretched adding to the increasing burden of cancer in the country [4,7]. This often results in the burden of care falling upon caregivers [8].

Caregivers are often considered as ‘relatives, friends, and partners who have a significant relationship with and provide assistance (i.e. physical, emotional) to a patient with a life-threatening, incurable illness’ [9]. In Vietnam, as in many lower and middle income countries (LMICs), caregivers fulfil several crucial roles, from providing informational and emotional support, to assisting with medical procedures, providing financial support and navigating hospital and medical administration. Caregivers face numerous challenges in fulfilling these roles, while also supporting and caring for themselves in the process [10]. In part, this is because there are no embedded or widespread support services or networks for cancer caregivers in Vietnam.

As Vietnam undergoes an epidemiological transition from communicable diseases to non-communicable diseases, the nature and burden of caring is likely to change dramatically. It has been well documented that cancer creates unique and significant challenges for caregivers, including on caregivers’ physical, emotional and mental health [11]. Stress, loss of control, anxiety and depression are all commonly experienced by cancer caregivers [10]. Previous studies have also demonstrated that poor health of the caregiver may correlate to poorer health for patients [12,13].

Research on the needs of caregivers in Vietnam is limited. The few studies that exist report on the needs of Vietnamese caregivers looking after people with dementia and/or Alzheimer’s disease [14], stroke [15], and HIV [16,17]. Other studies have been conducted with Vietnamese caregivers living in North America [18,19]. To date, there are no studies on the unmet needs of caregivers of hospitalized cancer patients in Vietnam. This study sought to provide empirical evidence on the self-identified unmet needs of caregivers of inpatients in national oncology hospitals in Vietnam.

## Methods

### Study design and data collection

**Figure 1.**
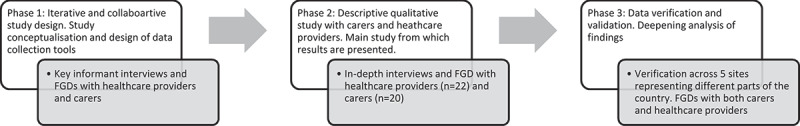
Study design

A three-stage approach was designed to inform the focus of the study, the development of data collection procedures, and to improve rigour and credibility through validating, verifying and triangulating data (Figure 1).

Phase 1 involved key informant interviews and focus groups discussions (FGDs) with healthcare providers and caregivers to understand context and inform the iterative and collaborative development of an interview schedule (see supplementary information). FGDs and in-depth interviews were conducted with caregivers and healthcare providers as part of Phase 2. Caregivers and healthcare providers participated in separate FGDs in order to reduce power imbalances impacting upon on our results. Data was collected in central, national-level oncology hospitals in both Hanoi and Ho Chi Minh City. Phase 3 involved FGDs in which the findings of the studies were presented to caregivers and healthcare providers for the purposes of verifying, validating and deepening the analysis of the results. Verification was conducted across five sites in Hanoi, Ho Chi Minh City, Hue, Da Nang and Can Tho.

Semi-structured FGDs and in-depth individual interviews were led by an experienced Vietnamese qualitative researcher, conducted in Vietnamese, and lasted between 60–90 minutes. All FGDs and in-depth interviews were partially translated simultaneously by a second Vietnamese researcher to enable non-Vietnamese members of the research team who were present during the FGDs and interviews to suggest relevant probes and additional questions with minimum interruption to the interviews. Probes were only offered at appropriate points within the interview, and when suggested by the lead Vietnamese interviewer.

### Setting, recruitment and study participants

Caregivers (n = 20) were recruited via convenience sampling within hospital settings in Hanoi K Hospital and the Ho Chi Minh Oncology Hospital. Healthcare providers (n = 22) in four departments of the two hospitals (radiology, surgery, chemotherapy and palliative care) were invited to participate in the study by administrative and medical contacts. Due to pragmatic constraints on both healthcare providers and caregivers time, both were offered the option of participating in either an in-depth interview or FGD. Four healthcare providers opted to participate via an in-depth interview option. All caregivers and other participants took part in FGDs. Caregivers were invited to participate in FGDs by healthcare workers at each hospital, and were recruited from different departments in the hospitals (e.g. radiology, surgery, chemotherapy, palliative care). Inclusion criteria were that participants were currently caring for cancer inpatients at the hospitals and were over the age 18. All healthcare providers and caregivers invited to participate and who met the inclusion criteria agreed and consented to taking part in the study. Recruitment and data collection for phase 2 took place in November 2018. All in-depth interviews and FGDs took place in either Hanoi K Hospital or Ho Chi Minh Oncology Hospital. Study participants were recruited until preliminary analysis of the data indicated that saturation had been reached for all major identified themes [20,21]. Each participant was provided with a small financial compensation for his or her time in contributing to the study.

### Data analysis

The ‘voice’ of caregivers is prioritised within the analysis, and is complemented and triangulated [22,23] with data from key informants and stakeholders such as doctors and other healthcare providers. These results were corroborated with data gathered through dissemination and verification in-depth interviews (phase 3). All in-depth interviews and FGDs were transcribed verbatim in Vietnamese and fully translated into English for analysis by the whole team.

Thematic content analysis was conducted [24] and an inductive codebook was collaboratively created based on the themes that emerged from initial independent readings. Analysis was conducted according to Braun and Clarke (2006) (familiarisation; coding; generation of initial themes; reviewing; refining and defining). All FGDs and in-depth interviews were initially analysed individually. No major differences in themes or between groups was identified. As such, data from different sources was then combined for analysis. Results were discussed extensively in both face-to-face and online meetings to improve rigour and reliability. Vietnamese members of the research team travelled to the UK for 10 days to contribute in-person to the analysis of the data. This face-to-face time between members of the multidisciplinary and international team allowed greater discussion, triangulation of results and key themes, and improved rigour within the analysis of results.

## Results

Caregivers (n = 20) were predominately female (65%), aged between 29–72 years old, with all caregivers with one exception being direct family members (e.g. parents, siblings, children and family by marriage). Cancer sites included breast, colorectal, oesophageal, stomach, and ovarian. HCPs (n = 22) represented a broad range of medical staff from departments of surgery, radiotherapy, palliation, nursing, nutrition and social work, and included staff in both senior and junior positions. With the exception of two caregivers within our key informant interviews (phase 1) and one in our main study (phase 2), all caregivers were informal and not paid.

Caregivers across our study communicated significant levels of need and that they had limited or no support across a range of domains. Equally, the lack of and need for psychosocial supports for caregivers was identified as critical by health care professionals. Four key areas requiring support were identified from the data. Unmet needs for caregivers in Vietnam were described related to the challenge of providing long-term care in hospital and inpatient environments. Such challenges related to requiring accessible and comfortable facilities, accommodation, and finance. Other reported needs related to information on cancer, cancer pathways, treatment, and nutrition; emotional support needs; and training on how to provide care during treatment and recovery. These needs were identified across all three participant groups with little variation and confirmed during phase 3 of the study in which the analysis was validated and deepened without new themes emerging.

### Challenge in providing long-term care in hospitals

Both caregivers (C) and health care providers (HCP) described the hospital/inpatient context as challenging and difficult. Caregivers and HCP noted the heavily overcrowded nature of centralised oncology hospitals, resulting in patients often sharing beds and caregivers sleeping in corridors or general areas of the hospital. Participants in our study acknowledged that caregivers contribute to this overcrowding. Caregivers are expected to remain bedside to support the needs of patients, yet are often asked to leave the ward during working hours. Given the extensive nature of tasks that caregivers are responsible for, many caregivers reported feeling resentful at being asked to leave their loved one’s bedside for fear the patient would be neglected if they were not there to provide support:

My wife has not been able to walk since the surgery. In the days after that, I had to help her to use the toilet and to feed her, and I was still asked to get out (of the ward). Sometimes I got mad. I said, “Now you don’t have anyone to care for the patient, but you still throw us out? We do not want to lie here, but we have to. If we are not here, who’s holding the bedpan?” Do the doctors and nurses ever take the bedpan to the patients? (C/HN)

Many caregivers and HCP reported that both caregivers and patients must travel long distances to reach national-level oncology hospitals in Hanoi (HN) or Ho Chi Minh City (HCMC). This results in difficulties for caregivers who must find affordable accommodation near the hospital for the duration of the patient’s treatment. Caregivers described situations in which they had to share motel rooms with other caregivers or stay in the corridors and communal areas of the hospital when asked to leave the wards. Often caregivers slept next to or under the patient’s beds when they were in the wards. Some caregivers staying in nearby motels described fearing for their safety; others noted the risk of having their possessions stolen if they slept in the hospital. These challenges created stress, fatigue and anxiety that compounded the already significant burden of caring for someone with cancer:

The first time I spent up to tens of million for renting a motel room out there. The first days, I didn’t know how to find a cheap motel. I came to a place up there and it cost me 300,000 VND per day (13 USD). I was shocked. It cost me a million [dong] for three days, without counting food costs. (C/HN)

Family members are not allowed to stay in the ward. They can only stay in the ward when needed. Family members have to take a mat to sleep outside in the corridor, and each time we come here for 25-30 days. I am the main caretaker. I sleep like that. I have aches and pains but I have to suffer. I am tired. (C/HCMC)

Economic challenges relating to the aforementioned accommodation and travel costs were additionally described as adding to the already difficult economic pressures experienced for families dealing with a cancer diagnosis. The stresses associated with economic challenges (from cost of treatment, indirect costs, and loss of income) from cancer diagnosis and treatment were highlighted by caregivers in this study:

My mom gets car sick, so she could not go by bus. Each time she went to Hanoi we had to pay 4 million [dong] for a taxi and 2 million [dong] for the train. (C/HN)

I think that most of the cancer patients that come here are from the provinces [rural areas]. We face difficulties in [terms of] travelling time. And worse is the financial problems. The majority of cancer patients are [living] in remote provinces and live in difficult conditions. We eat charity rice. We eat charity porridge. (C/HN)

### A lack of knowledge of cancer, cancer pathways, and treatment

Many participants described limited knowledge about cancer, and thus having a number of informational needs. Caregivers required information related to diagnosis, expectations for treatment, nutrition and traditional medicine, and information they needed as caregivers to both support themselves and on accommodation and places to stay near the hospital. This lack of information or knowledge about cancer was suggested to add to the caregiver burden as they felt confused and unsure if they were doing the right thing:

Even though we are family members, our knowledge about the field of cancer is still very limited. Now I have to take care of nutrition and psychological health. My experience of patient care is very limited. (C/HN)

Information needs for the patient were normally prioritised by caregivers in the study. Caregivers wanted information on a wide range of factors, from the aetiology of cancer, to information on the ‘best’ hospitals and doctors, to information on probability of survival:

We needed to choose a place to get treatment. For cancer, there are many places, but how do we find the best and most suitable? That is very important because there are many hospitals that have the ability to treat – like here, they focus on cancer treatment. (C/HCMC)

Caregivers often requested simple information such as the stage of the patient’s diagnosis and what this meant. They often reported a lack of information and communication between healthcare providers, caregivers and patients. This was often attributed to the lack of time that HCPs had for in-depth conversations. This problem is potentially exacerbated by patients relying on internet sources for information about cancer, and not knowing which sources to trust and which sources were accurate:

Basically, I’m not a person in the field [of cancer] so I do not have the knowledge. But the information on the Internet is too wide, so the knowledge we learn from the Internet is difficult to apply for the care of our family patients – what to eat, what not to eat, is milk allowed or not?. (C/HCMC)

Information from different sources is not correct, thus the readers have the wrong understanding. So, when the patient asks questions, the doctor will explain the information to the patient accurately. But the patient will not accept the explanations of the doctor because what the doctor has said is different from what they have heard or read. (HCP/HCMC)

Caregivers reported a high level of need regarding nutritional information. Caregivers described a feeling of confusion or uncertainty relating to what they should be feeding the patient and when. Caregivers wanted to know what food they should provide (caregivers provide all meals to patients) to help maximise patients’ treatment outcomes and recovery. Specific dietary information was requested for different types of cancer, different stages of diagnosis, and different stages of the treatment process (e.g. food before and after surgery, food during chemotherapy, etc.):

The doctor is not able to advise you carefully about nutrition; they are only consulted about the [patient’s] drugs or the daily necessities. I had to search the Internet, but the information from internet is not the same. (C/HN)

Information needs to support the caregiver focused around training and on providing practical information on how to navigate the hospital administration, how to find cheap and comfortable accommodation, and how to support their emotional and mental health. It was suggested that resources such as booklets and online information should be created and provided to caregivers when they arrive in the hospitals.

### The emotional impact of care

Caregivers described a wide range of emotional and psychological needs and a lack of specialised services available to help them cope with the strain and emotional impact of caring. Caregivers described feelings of stress, shock (at the diagnosis), fatigue, grief, and sadness. These challenges affected their physical and psychological health. HCPs additionally observed similar emotions and strains, and described how patients sometimes worry that their caregiver will abandon them due to the stresses they experience in their role:

I went to the second floor to cry hourly because she (her mother) was diagnosed at the late stage. [But] my mother is so old she may die on the operating table. So even with such surgery she cannot live long, either. It is very miserable psychologically. (C/HN)

Usually in the late stages, the patient is afraid of being abandoned because many caregivers have been helping for a long time. They are tired, and could not afford to pay anymore and they will abandon the patients. Patients are really afraid of that. (HCP/HCMC)

Caregivers additionally reported stresses upon relationships between caregivers and the patient, and of the lack of support groups and communities to address this problem. These emotional stresses resulted in relationship difficulties or spousal conflict which caregivers found difficult to manage. Caregivers noted that they received no formal support to manage these concerns. Most support for caregivers came from their peers on the wards or in motels close to the hospitals rather than from formalised services. Spousal conflict and challenges in decision making were frequently described:

Patients often feel uncomfortable. Husbands and wives also fight and argue … so we need to calm them down … Some couples in the ward fight and swear at each other. I told the wife to forgive the husband [referring to the patient] because he has a disease and is under pressure. He refused to take drugs, swore and asked for death. So [I told his wife], “You have to say nice words, persuade him. (C/HCMC)

### Training needs to support effective care

Caregivers indicated a need for training and preparation for being a caregiver, particularly around certain clinical procedures. This included information on providing how to care for their family member and how best to support them during their treatment and recovery. Due to the crowded nature of hospitals, caregivers described having to undertake roles in providing medical treatment, for example holding chemotherapy bags, holding IV fluids, and changing dressings on wounds. Caregivers reported having little medical knowledge and requiring specific training to fulfil these roles, particularly given that they would need to continue some of these roles when the patient is discharged:

By the end of the week, the doctors and nurses are busy. If I call [for one of them], it takes them a very long time to get here because they are busy taking care of many people. Sometimes I have to learn how to do it myself. Sometimes I even want to learn the way to get the veins myself. They are so busy that sometimes they make mistakes. (C/HN)

I have asked the doctor very carefully, so when the doctor came, I asked him what is the best way to stop bleeding and how to treat hemorrhage. The doctor told us some methods, so now it’s stable. But the thing is when I get home, I don’t know how to treat. I have to ask the doctor how to stop bleeding temporarily so that I can bring the patient here. (C/HCMC)

Along with specific and appropriate training, caregivers highlighted the need for clearer signposting to different services and sources of information in the hospital. While some supportive resources and services have been developed (such as information sessions, online groups, leaflets and posters), often caregivers did not know how to access these resources. HCPs recognized the value of having caregivers who were trained and prepared, and suggested that training could be provided to caregivers to better support them. Although paid caregiving is uncommon in Vietnam, some HCPs suggested this was a possible area for growth.

## Discussion

Cancer caregivers in Vietnam are experiencing multiple and complex unmet needs. These unmet needs greatly affect caregiver’s health and influence the extent to which they can adequately care for patients. Needs are material, informational, and emotional. Despite the high level of burden, support for caregivers is limited. There is a lack of psycho-oncology and supportive care in Vietnam, and resources and expertise in this area are required.

Caring and family are largely synonymous and inseparable in Vietnam. One study exploring the needs of Vietnamese caregivers in Canada described the obligation to care for family members as the norm, stating, *“It’s like eating, you just do it’* [19]. Our study found similar themes and highlighted the importance of thinking about the needs of caregivers both individually and in terms of supporting the care of patients.

The nature and burden of caring in Vietnam has likely shifted dramatically within a short period. For much of history, caring would have been in response to (mainly) infectious and communicable disease. Such disease required care for short intense periods. Cancer, and many non-communicable diseases, change this model. The number of cancer cases in Vietnam is steadily increasing [4,25], however, how caregiving is conceptualised remains largely unchanged. Caregivers are still expected to be present and central in the care of their family member, and are expected to sacrifice time, work, and often their own health in the process.

As shown within this, and many other studies [26], cancer requires time-intensive care for prolonged periods in specialised and centralised oncology hospitals. Cancer remains poorly understood in Vietnam, and there is low awareness concerning what to expect within both treatment and care [5]. Cancer treatments are invasive and often permanent. Cancer is also expensive to diagnose and treat [27,28]. All these factors make the experience of caring for someone with cancer in Vietnam (largely) new. New forms of support have yet to be developed despite this need, and despite the changing burden of care, and as a result caregivers are living with a high level of sustained and unmet needs. Patient support networks are becoming increasingly established for people diagnosed with cancer in Vietnam. Such clubs are reported by patients to have provided significant emotional support and solidarity as patients navigate difficult shared experiences [7]. Such clubs have not yet been established for caregivers, and may represent an area for future studies and interventions.

Information resources (both online and physical) along with the creation of communal rooms for caregivers to stay in when they have to leave the wards, should be considered. Such pragmatic, low cost interventions have the potential to greatly reduce challenges experienced by caregivers, and should be explored within future interventions. Training courses for informal caregivers may support capacity-building for caregivers and may also provide a point of contact for face-to-face dissemination of information specific to caring. Interventions testing training for dementia caregivers have been previously trailed with success [29], and could be explored in relation to the specific needs of cancer caregivers.

## Clinical implications

This study has implications particularly around how clinical information is communicated to caregivers, and how appropriate training may be provided to caregivers for them to best care for family members both in hospitals and at home. Comprehensive psychosocial and supportive care is required for caregivers of someone with a cancer diagnosis. Support needs to be extended beyond medical treatment to provide holistic care for patients and their affected families.

## Study limitations

As this study was conducted in hospital settings it is likely not reflective of specific or different needs that caregivers experience at home and in the community. In-depth research that also explores different needs for people caring in the community should be explored. Given difficulties in accessing oncology hospitals due to location and costs, research on needs to the community may access caregivers with the most pronounced challenges. As with any study using translated transcripts, there is a risk of losing meaning or nuance. We sought to offset this through the extensive face-to-face time analysing the transcripts, and the extensive involvement of the Vietnamese team members in checking translated transcripts against original transcripts. Offering a small amount of financial compensation for participation in research is a norm in Vietnam, and the impact this may have had on participation should be considered in the interpretation of the results of the study.

## Conclusions

This paper highlights the specific and unique unmet needs of caregivers looking after cancer patients in Vietnam. Caregivers described the significant challenges they face in terms of lacking accurate information, requiring emotional support, training and signposting of different services. Interventions to test the effectiveness, acceptability, cost-effectiveness and scalability of physical and online informational and supportive resources would be an invaluable next step in supporting caregivers manage both the health of patients as well as supporting their own health and wellbeing. Integration and increased visibility and recognition of caregivers within the health care system would likely benefit both caregivers and the people they care for.

## Supplementary Material

Supplemental Material

## Data Availability

The datasets used and/or analysed during the current study are available from the corresponding author on reasonable request.

## Paper context

Little published research exists on the needs of people caring for someone with cancer in Vietnam. This important group experience critical challenges and require support in their vital roles in caring for people with a cancer diagnosis. Understanding these needs is the first stage in developing appropriate solutions and support.
