# Supplementary material for: A qualitative study on the needs of cancer caregivers in Vietnam
Source: Glob Health Action. 2021 Aug 26;14(1):1961403. doi: 10.1080/16549716.2021.1961403 (PMC8405114; doi:10.1080/16549716.2021.1961403)
Supplement: Supplemental Material [file ZGHA_A_1961403_SM4732.docx]

| **No** | **Item** | **Guide questions/description** |
| --- | --- | --- |
| **Domain 1: Research team and reflexivity** |  |  |
| Personal Characteristics |  |  |
| 1. | Interviewer/facilitator | Which author/s conducted the interview or focus group?  HTH and NLPH led data collection in focus group discussions and semi-structured interviews. OS and CJ contributed to data collection through asking questions within FGDs and interviews. |
| 2. | Credentials | What were the researcher's credentials? *E.g. PhD, MD*  HTH, OS = PhD |
| 3. | Occupation | What was their occupation at the time of the study?  Lecturers |
| 4. | Gender | Was the researcher male or female?  HTH, NLPH, OS = female. CJ = male. |
| 5. | Experience and training | What experience or training did the researcher have?  HTH and OS led the study, both with 10-15 years experience in qualitative research |
| Relationship with participants |  |  |
| 6. | Relationship established | Was a relationship established prior to study commencement?  Relationships established with HCPs and with carers to inform study design. |
| 7. | Participant knowledge of the interviewer | What did the participants know about the researcher? e*.g. personal goals, reasons for doing the research*  Fully informed about study and how it would be used (see – Methodology and Ethics statements) |
| 8. | Interviewer characteristics | What characteristics were reported about the interviewer/facilitator? e.g. *Bias, assumptions, reasons and interests in the research topic*  Gender and nationality reported. (Contributors statement and Methodology) |
| **Domain 2: study design** |  |  |
| Theoretical framework |  |  |
| 9. | Methodological orientation and Theory | What methodological orientation was stated to underpin the study? *e.g. grounded theory, discourse analysis, ethnography, phenomenology, content analysis*  Descriptive qualitative study |
| Participant selection |  |  |
| 10. | Sampling | How were participants selected? *e.g. purposive, convenience, consecutive, snowball*  Convenience (Methodology) |
| 11. | Method of approach | How were participants approached? e*.g. face-to-face, telephone, mail, email*  Via an intermediary medical administration contact in each hospital (Pg. 3 - Methodology) |
| 12. | Sample size | How many participants were in the study?  44 in main study |
| 13. | Non-participation | How many people refused to participate or dropped out? Reasons?  None |
| Setting |  |  |
| 14. | Setting of data collection | Where was the data collected? e*.g. home, clinic, workplace*  Hospital meeting rooms – for convenience as all participants were active carers for in-patients. |
| 15. | Presence of non-participants | Was anyone else present besides the participants and researchers?  No |
| 16. | Description of sample | What are the important characteristics of the sample? *e.g. demographic data, date*  The sample of carers (n=20) (Table 1.) was predominately female (65%), aged between 29-72 years old, with all carers with one exception being direct family members (e.g. parents, siblings, children and family by marriage). Cancer sites included breast, colorectal, oesophageal, stomach, and ovarian. HCPs (n=22) represented a broad range of medical staff from departments of surgery, radiotherapy, palliation, nursing, nutrition and social work, and included staff in both senior and junior positions. |
| Data collection |  |  |
| 17. | Interview guide | Were questions, prompts, guides provided by the authors? Was it pilot tested?  Yes guidelines provided. Not pilot tested, however key informant interviews were conducted prior that informed the approach, study design, and interview guide development |
| 18. | Repeat interviews | Were repeat interviews carried out? If yes, how many?  No |
| 19. | Audio/visual recording | Did the research use audio or visual recording to collect the data?  Audio |
| 20. | Field notes | Were field notes made during and/or after the interview or focus group?  Yes, by all researchers present. |
| 21. | Duration | What was the duration of the interviews or focus group?  45-90mins each |
| 22. | Data saturation | Was data saturation discussed?  Unclear (Highlighted in Discussion) |
| 23. | Transcripts returned | Were transcripts returned to participants for comment and/or correction?  No – but the third phase of study was used to disseminate, validate and verify findings |
| **Domain 3: analysis and findings** |  |  |
| Data analysis |  |  |
| 24. | Number of data coders | How many data coders coded the data?  6 |
| 25. | Description of the coding tree | Did authors provide a description of the coding tree?  Can be provided on request, but discussed briefly in Data Analysis |
| 26. | Derivation of themes | Were themes identified in advance or derived from the data?  Derived from data |
| 27. | Software | What software, if applicable, was used to manage the data?  n/a |
| 28. | Participant checking | Did participants provide feedback on the findings?  Yes – during phase 3 – validation and verification of data |
| Reporting |  |  |
| 29. | Quotations presented | Were participant quotations presented to illustrate the themes / findings? Was each quotation identified? e*.g. participant number*  Yes (Throughout Results) |
| 30. | Data and findings consistent | Was there consistency between the data presented and the findings?  Yes |
| 31. | Clarity of major themes | Were major themes clearly presented in the findings?  Yes |
| 32. | Clarity of minor themes | Is there a description of diverse cases or discussion of minor themes?  Yes |
